# [Retracted] Adipose extracellular matrix promotes skin wound healing by inducing the differentiation of adipose-derived stem cells into fibroblasts

**DOI:** 10.3892/ijmm.2024.5430

**Published:** 2024-09-23

**Authors:** Zhi-Qiang Zhou, Yi Chen, Mi Chai, Ran Tao, Yong-Hong Lei, Yi-Qing Jia, Jun Shu, Jing Ren, Guo Li, Wen-Xin Wei, Yu-Di Han, Yan Han

Int J Mol Med 43: 890-900, 2019; DOI: 10.3892/ijmm.2018.4006

Following the publication of this paper, it was drawn to the Editor's attention by a concerned reader that certain of the immunohistochemical images shown in Fig. 2B and C on p. 896 were strikingly similar to data appearing in different form in other articles written by different authors at different research institutes that had already been published before this paper was received at *International Journal of Molecular Medicine* (several of which have been retracted). Moreover, the flow-cytometric data shown in Fig. 2A appeared to be potentially anomalous.

In view of the fact that the abovementioned data had already apparently been published prior to the submission of this paper to *International Journal of Molecular Medicine*, the Editor has decided that the article should be retracted from the Journal. The authors were asked for an explanation to account for these concerns, but the Editorial Office did not receive a reply. The Editor apologizes to the readership for any inconvenience caused.

